# Long-Term Increase of Radiographic Damage and Disability in Patients with RA in Relation to Disease Duration in the Era of Biologics. Results from the SCQM Cohort

**DOI:** 10.3390/jcm7030057

**Published:** 2018-03-13

**Authors:** Katja Heinimann, Johannes von Kempis, Rafael Sauter, Michael Schiff, Tuulikki Sokka-Isler, Hendrik Schulze-Koops, Rüdiger Müller

**Affiliations:** 1 Division of Rheumatology, Immunology and Rehabilitation, Kantonsspital St. Gallen, 9007 St. Gallen, Switzerland; katja@heinimann.ch (K.H.); johannes.vonkempis@kssg.ch (J.v.K.); 2 Clinical Trials Unit, Kantonsspital St. Gallen, 9007 St. Gallen, Switzerland; rafael.sauter@gmail.com; 3 School of Medicine, University of Colorado, Denver, CO 80111, USA; michael.schiff@me.com; 4 Faculty of Health Sciences, Jyvaskyla Central Hospital, University of Eastern Finland, 40620 Jyvaskyla, Finland; tuulikki.sokka-isler@ksshp.fi; 5 Division of Rheumatology and Clinical Immunology, Department of Internal Medicine IV, Ludwig-Maximilians-University Munich, Pettenkoferstr. 8a, 80336 Munich, Germany; Hendrik.Schulze-Koops@med.uni-muenchen.de

**Keywords:** disease duration, rheumatoid arthritis, DAS28, HAQ-DI, radiographic destruction, Ratingen score, rheumatoid factor, ACPA

## Abstract

Objectives: There is little information on the relation between disease duration, disability and radiographic outcome since the introduction of biologics into the therapy of rheumatoid arthritis (RA). No long -term cohort studies have been conducted on this subject so far. To analyse radiographic damage, disability, and disease activity in RA-patients dependent on disease duration in the Swiss national RA cohort (SCQM). Methods: The primary outcome was the association between the radiographic destruction, assessed by Ratingen scores, and disease duration. All patients with at least one clinical visit were analysed with polynomial and multiple negative binomial models. Results: The disease duration in the 8678 patients with available radiographs analysed ranged between less than 1 and more than 65 years (median 8.3). Disease duration and radiographic destruction were significantly associated with an average increase of Ratingen scores by 8.3% per year. Apart from disease duration, positive rheumatoid factor was the strongest predictor for radiographic destruction. While DAS28-scores remained stable in patients with a disease duration of more than 5 years (median DAS28 2.8), HAQ-DI scores increased continuously by 0.018 for each additional year. Conclusion: In this RA cohort, patients show a continuous increase of articular destruction and physical disability in parallel with disease duration. Even when nowadays a satisfactory control of disease activity can be achieved in most patients, RA remains a destructive disease leading to joint destruction and physical disability in many patients.

## 1. Introduction

Rheumatoid arthritis (RA) is a chronic inflammatory disease with a variable, but mostly progressive course over many years after its onset. The disease is associated with progressive joint destruction [1], a decreased quality of life [2,3], systemic manifestations, and comorbidities [4]. The financial and social burden of this destructive disease has been described in many analyses [3,5,6,7]. 

Several prospective studies found the radiographic joint destruction and disability to progress continuously over time [1,8]. Wolfe et al. [1] analysed 296 recent onset RA patients from 1973 to 1993. They found an average of 1.0 newly eroded joints per year and patient and a yearly increase in Sharp score by 4.5 points. Scott [8] reviewed the radiographic progression in therapeutic clinical trials published between 1995 and 2002. He described an average radiographic progression (Sharp or Larsen scores) of 2% of maximal possible damage per year, despite treatment with conventional synthetic DMARDs in the majority of treatment arms.

Treatment strategies have drastically improved over the last decades. Methotrexate [9] and tight control strategies [10,11] became standard of care in the 1980s and 1990s. With the introduction of biologic agents [12] the spectrum of therapeutic options broadened and a new era began [13].

In clinical trials, a great improvement of disease activity, radiographic course, and functional ability was found under biologic treatment as compared to methotrexate monotherapy [12,14,15,16]. This positive effect on functional disability was confirmed in various cohort studies [17,18] outside the setting of clinical trials. However, other cohort studies could not reproduce this effect [6,19]. The general observation among rheumatologists, that radiographic progression has become milder over the last decades [20,21] was found to be attributable to the treatment with DMARDs as well [22]. To our knowledge, there are no observations of radiographic damage in RA in relation to the disease duration in the era of biologic treatment.

The aim of this study was to investigate the radiographic destruction, disability, and disease activity depending on disease duration in this new era of available biologic treatment. This was analysed cross-sectionally within the SCQM (Swiss Clinical Quality Management) cohort using patient data collected over the last 20 years.

## 2. Methods

### 2.1. Study Population and Design

This cohort study analyses data from the SCQM, a registry run by office or hospital-based rheumatologists since 1996. Inclusion criteria for the analysis were a diagnosis of RA by a rheumatologist. Clinicians participating in the SCQM provide clinical patient data and radiographs of hand and feet on a regular basis. A central reader from the SCQM assesses these radiographs for destruction using Ratingen erosion scores (range 0–190) [23]. A more detailed description of the SCQM registry is described elsewhere [24].

### 2.2. Exposure of Interest

Patient data from the last visit available in the database were analysed cross-sectionally dependent on their individual disease duration. The disease duration was defined as the time between the first diagnosis and the last follow up visit documented in the SCQM database. Our analysis takes into account data entered into the SCQM database between 1998 and November 2015.

### 2.3. Inclusion/Exclusion Criteria

RA patients with one or more clinical visits available in the database were included in the analysis. We excluded patients with missing information on disease duration. All outcomes were exclusively analysed based on available data. Missing observations were considered to be missing at random and, therefore, no assumptions for these missing data were made.

### 2.4. Outcome Parameters

The association between the radiographic destruction, assessed by Ratingen erosion scores at the last visit, and the disease duration, was the primary outcome of interest. Disease activity and disability in dependence of disease duration, served as secondary outcomes and were evaluated using DAS28 [25] and HAQ-DI scores [26], respectively.

We hypothesised, that disease duration, age, gender, rheumatoid factor (RF), ACPA (anti citrullinated peptide antibodies), and therapeutic status regarding the use of anti-TNF drugs and methotrexate (MTX), explain a significant amount of the variability of the investigated outcomes.

### 2.5. Statistical Analysis

The possible non-linear association between our outcomes of interest (Ratingen erosion score, HAQ-DI, DAS28) and the disease duration was investigated with an exploratory, non-parametric local polynomial regression (loess function in R-package stats) using a smoothing parameter of 0.5, which corresponds to bandwidth of six months of disease duration. The results are illustrated as smoothed time trends with a 95% confidence interval.

Due to possible confounding factors (age, gender, RF, ACPA, MTX exposure and anti-TNF drug exposure), the hypothesised dependency between the response variables (Ratingen erosion score, HAQ-DI and DAS28) and the disease duration was also investigated with parametric regression models. As the Ratingen score is a count variable which is characterized by strong over dispersion (mean 24.3, variance 923.8), a multiple negative binomial regression model was applied to model this outcome. The expected dependencies between HAQ-DI or DAS28 and disease duration were analysed with multiple linear regression models. The latter outcome was examined with a regression model that reflects the initial decrease and following increase in the outcome (DAS28 and HAQ) by the use of two time-dependent regression parameters: one for the disease duration below and the second for the disease duration above five years.

Additionally, we investigated correlations between (a) Ratingen scores and HAQ-DI, as well as (b) DAS28 scores and HAQ-DI. Since these variables did not follow a normal distribution, we used Kendall’s Rank correlation to assess any form of dependency.

All analyses were performed with the R statistics package (R Foundation, Vienna, Austria, version 3.2.3, R Core Team 2013). We did the negative binomial regression analysis with the procedures implemented in the MASS library (Venables and Ripley, New York City, NY, USA, version 7.3.45, 2002).

## 3. Results

### 3.1. Patients

The analysis was based on 52,753 records of 8678 RA patients available in the SCQM cohort (status November 2015). Of these we excluded 823 patients due to missing information on disease duration. Selecting patients with information available for each investigated outcome, the analysis comprised 6361 patients for Ratingen scores, 6365 patients for DAS28 scores and 5795 patients for HAQ-DI scores.

### 3.2. Distribution of Disease Duration

**Figure 1:**
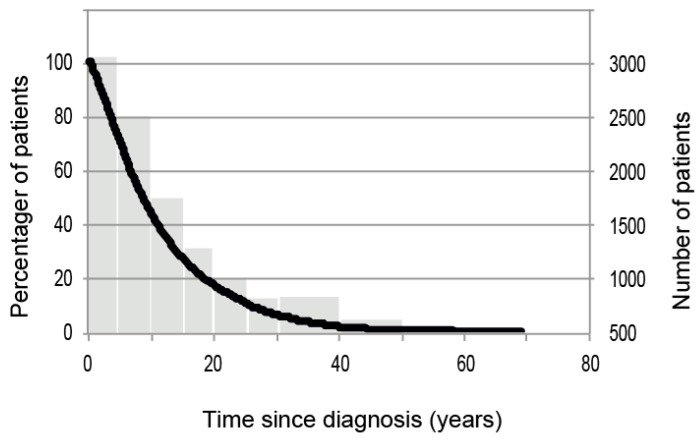
Patient distribution according to disease duration illustrated as individual data (black line) and as a histogram representing the subgroups described in Table 1.

The disease duration ranged between 4 months and 68.8 years (mean 11 years). With increasing disease duration, we found a logarithmic decline in patient numbers in our cohort. Based on a regression model with a constant linear decline on the logarithmic scale, the patient population is estimated to decline by 9.24% per year (Figure 1, R^2^ = 0.99). There were 462 patients with a disease duration of more than 30 years. Please refer to Table 1 for a more detailed depiction of the patient distribution.

### 3.3. Demographical Data

The mean age of the patients analysed was 59.9 years. The age increased from an average of 57.0 years in patients with a disease duration of less than 5 years, to 71.5 years in patients with a disease duration of over 40 years.

The majority of patients analysed was female (74.4%). A positive rheumatoid factor was present in 70.8% of patients, ACPA in 62.2% of patients (both analysed on patients with available data). Both antibodies were less frequent in patients with recent onset RA and increased with longer disease duration.

Past or current exposure to MTX was reported for 81.9 % of the patients. There was a slightly lower rate of MTX exposure in patients with a disease duration of more than 20 years. The rate of patients pre-exposed to anti-TNF drugs increased with time from 42.4% in patients with a disease duration of less than 5 years, to between 60 and 70% of patients with a longer disease duration. 27.5% of all patients had a history of other biologic treatment than TNF antagonists (data not shown).

### 3.4. Primary Outcome

**Figure 2:**
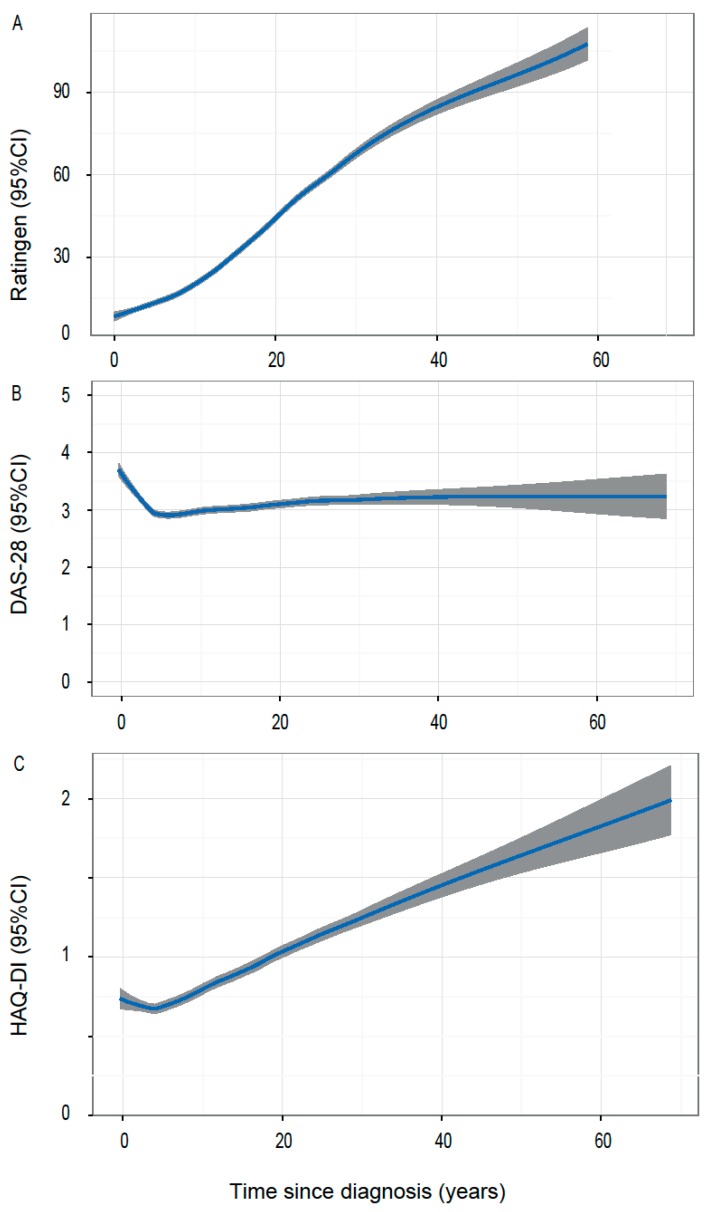
Smoothed time trends for radiographic damage, disease activity, and patient centred outcome: (**A**) Ratingen scores, (**B**) DAS28 scores, and (**C**) Health assessment questionnaire-disability index (HAQ-DI) scores in dependence of disease duration as modelled by local polynomial regression using a smoothing parameter of 0.5. Results are shown with 95% confidence intervals (CI, grey shade).

**Figure 3:**
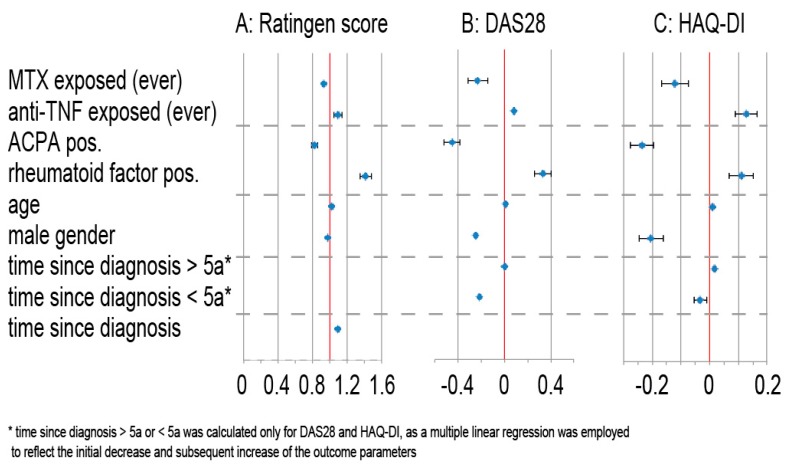
(Column **A**) Results for the negative binomial regression model with the Ratingen score as outcome. The estimate for each of the parameters indicates the percentage change in Ratingen score associated with a change of said parameter. While most variables included in the model are binary, for the continuous variables, such as time since diagnosis and age, the estimates refer to an increase of the parameter by 1 unit. Values greater than 1 indicate a positive association, smaller than 1 indicate a negative association. Results for the linear regression models for (Column **B**) the DAS28 score and (Column **C**) the HAQ-DI. The estimate for each of the parameters indicates how much the score changes in absolute, if only this parameter changes. While most variables included in the model are binary, for the continuous variables, such as time since diagnosis and age, the estimates refer to an increase of the parameter by 1 unit. Values greater than 0 indicate a positive association, smaller than 0 indicate a negative association.

The local polynomial regression model for Ratingen scores revealed a continuous rise of radiographic destruction with increasing disease duration (Figure 2). This increase of the Ratingen score was less pronounced during the first few years of the disease. The negative binomial regression model demonstrates an increase of the radiographic destruction by 8.6% (Figure 2A) for each additional year of disease. Apart from time, a positive rheumatoid factor and pre-exposure to anti-TNF drugs were the strongest predictors for joint damage. ACPA positivity and MTX pre-exposure, on the other hand, were associated with lower Ratingen scores (Figure 3).

### 3.5. Secondary Outcomes

In patients with disease duration of 5 years or less, the DAS28 score, as well as HAQ-DI scores, decreased with time (Figure 2B,C). While the majority of patients with a disease duration of more than 5 years remained in low disease activity (Figure 2B), there was a steady increase of HAQ-DI for each additional year of disease duration (Figure 2C).

The multiple linear regression model confirmed a time-dependent decrease of the HAQ-DI during the first 5 years since diagnosis (*p* = 0.004) and a time-dependent increase when disease duration was greater than 5 years (*p* = 0.0001) with an increase of the HAQ-DI by 0.175 points for each year. The parameter estimates for the possible confounders are shown in Figure 3, third column. Positive rheumatoid factors increased the HAQ-DI by 0.11, while male gender and ACPA positivity resulted in lower HAQ-DI scores (by 0.20 and 0.24 respectively). MTX pre-exposure was associated with a HAQ-DI score of 0.12 points less, while the HAQ-DI scores of patients under past or current treatment with TNF-antagonists were higher by 0.13 points. All variables were significant on a level of <0.0001.

With regard to disease activity, a decrease of DAS28 by 0.22 points was apparent within the first five years of disease (Figure 3, second column). A positive rheumatoid factor was associated with a DAS28 score that was 0.33 points higher, while positive ACPA is associated with DAS28-scores that are 0.45 points lower. These variables were significant on a level of <0.0001. Age had only a minimal effect on disease activity, while TNF antagonist-exposure, and time since diagnosis more than 5 years, had no significant effect at all.

**Figure 4:**
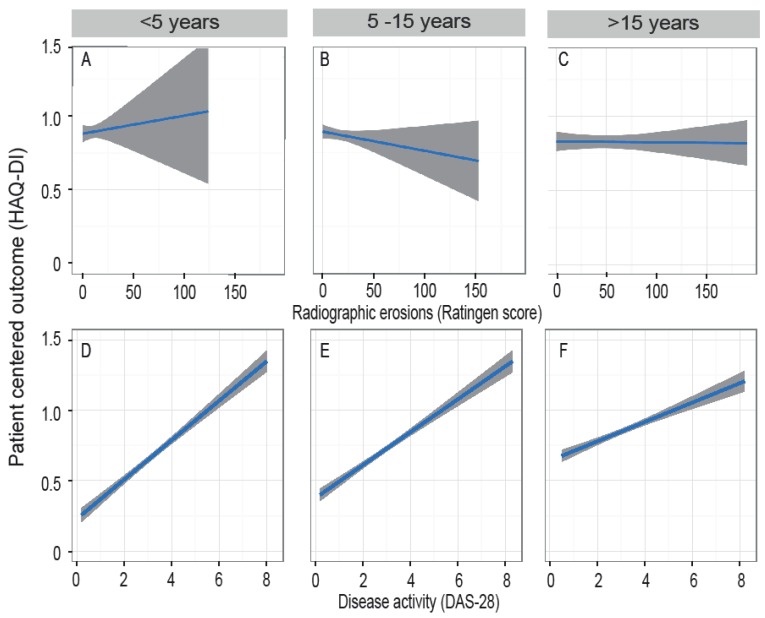
Correlation of HAQ-DI with radiographic destruction and disease activity stratified according to disease duration: The dependency of the HAQ-DI score from (**A**–**C**) Ratingen scores and (**D**–**F**) DAS28 scores for patients with a disease duration of <5 years (**A**,**D**) 5-15 years (**B**,**E**), and >15 years (**C**,**F**) are modelled by local polynomial regression fittings using a smoothness parameter of 0.1. Results are shown with 95%-CI (grey shade).

As both Ratingen scores (Figure 2A) and HAQ-DI scores (Figure 2C) increased with disease duration, we analysed whether Ratingen scores correlated with HAQ-DI scores in this cohort. This calculation included 5795 patients for the correlation between HAQ-DI and DAS28 scores, and 3966 patients for the correlation between HAQ-DI and Ratingen scores. The rank correlation according to Kendall showed an overall correlation between HAQ-DI and DAS28 of 0.31. This correlation was the strongest for patients with disease duration of less than 5 years (0.37, Figure 4A) and decreased with time (0.30 for disease duration 5–15 years, Figure 4B, and 0.23 for disease duration of >15 years, Figure 4C). No relevant correlation between HAQ-DI and Ratingen scores could be found (Figure 4D–F).

## 4. Discussion

This cross-sectional cohort study analyses disease activity, radiographic joint destruction and disability in patients with RA depending on disease duration. Our results show that rheumatoid arthritis today is still a destructive disease with a high impact on functionality in daily life. This is of great importance, since these are direct predictors for work disability and mortality in RA [6,27].

### 4.1. The Extent of Radiographic Damage has Decreased in Comparison to Earlier Analyses

Our data show that with every year of disease duration the Ratingen score increased by 8.6%. In other words, the Ratingen score doubled within 8.25 years. In comparison, Wolfe et al. [1] found in 1998 an average of 1.0 newly eroded joints per year and patient. They described a 4.5 unit increase in Sharp score per year. Based on an estimated baseline Sharp score of four in this cohort, this results in almost a doubling of Sharp scores within the first year. In parallel, Scott et al [8] have reported in 2004 that patients reached 11% of maximal damage by 5 years and over 40% by 20 years. In our cohort, 11% of maximal damage (=Ratingen score of 20.9) was reached after 10 to 15 years of disease duration and 40% of maximal damage (=Ratingen score of 76), on a group level, only after more than 40 years of disease duration (data not shown). In conclusion, the rate of radiographic destruction in RA patients in our analysis was lower than compared to these historical cohorts. We think that this can be explained by the improved treatment options available today. In our cohort, 57.7% of the patients were exposed to TNF antagonists. However, patients with a longer disease duration were already suffering from RA prior to the introduction of biologic agents. Whether earlier treatment with biologic DMARDs in these patients would have resulted in a milder disease course with less radiographic destruction and better functional ability, remains speculative.

### 4.2. Radiographic Destruction and Use of TNF Antagonists

A disconnect between radiographic progression and disease activity under treatment with biologic agents has been discussed [28,29]. In the multiple negative binomial regression model (Figure 3A), we found pre-exposure to TNF antagonists to be associated with greater radiographic destruction. What at first sight seems like a negative effect of anti-TNF treatment on joint destruction, in our opinion reflects the characteristics of the patient population receiving biologic DMARDs: Its usually insufficient control of clinical activity or radiographic progress that leads rheumatologists to the escalation of treatment with a biologic, in our cohort usually a TNF antagonist, results in a selection of patients suffering from a more aggressive form of RA. The positive effect of a combination therapy with biologic DMARDs compared to Methotrexate alone has been demonstrated in many studies [12,14,15,16] and was not the subject of this investigation.

### 4.3. ACPA Positivity Associated with Milder Disease Course

In our analysis, ACPA positivity was a predictor for improved clinical outcomes: It was associated with less radiographic destruction, lower disease activity and less functional impairment. This stands in contrast to some studies, that found a positive ACPA profile to be associated with more severe radiographic damage and a higher disease activity [30,31]. More recent studies confirm our findings, that the presence of ACPA—in contrast to RF—is at least associated with lower disease activity [32].

### 4.4. Disconnect between Disability and Radiographic Destruction

In patients who have been suffering from RA for more than five years, data from our cohort show a continuous increase in HAQ-DI, as well as Ratingen scores over time. However, the implied presumption that the articular destruction, measured by the Ratingen score, might be responsible for the impairment in function cannot be confirmed by our data. We found no relevant correlation between Ratingen scores and HAQ-DI scores. This is in line with the findings of Sokka et al. [33], that there is only a weak correlation between radiographic destruction and HAQ-DI score. These authors showed that the HAQ-DI score is primarily explained by the patient’s global assessment of pain. This finding was reaffirmed by Moleenar et al. [34] in an analysis of 186 RA patients in remission, that found disease activity and patient’s global assessment of pain to be strongly associated with functional disability as analysed by HAQ-DI. The hypothesis that radiographic destruction is associated with a loss of functional ability at a later time of the disease, as suggested by Bombardier et al. [35], cannot be certificated in this cross-sectional analysis. It has been discussed that joint destruction of large joints correlates with HAQ-DI scores [36]. This finding cannot be assessed in our analysis as no information on the radiographic damage of large joints is collected in the SCQM cohort and the Ratingen score analyses erosions of hands and feet only. In our cohort, the HAQ-DI scores correlated with the DAS28 scores.

### 4.5. Extra-Articular Manifestations Increase in Parallel to Disease Duration

The number of patients suffering from extra-articular RA manifestations in our cohort also increased over time (Table 1), whereas the entity of the extra-articular manifestation is not differentiated in the SCQM database. While in recent onset patients (disease duration less than 5 years) just over half of the patient population is affected, there was a continuous increase over time, amounting to over 90% after 30 to 40 years of disease duration. These numbers are much higher than the prevalence of 10 to 20% reported in other studies [37,38]. Since therapeutic strategies for extra-articular RA manifestations have not been well established, it is important to keep out an eye for the long-term development of extra-articular manifestations, even if good control of joint inflammation has been achieved.

### 4.6. Strengths and Limitations

This study fills an important gap, as there have been no reports on long-term outcomes in patients with RA in relation to disease duration since the early 2000s, when biologics had just been introduced. With a patient population of almost 8000 patients, of which more than half have been exposed to TNF-antagonists, this patient cohort reflects the increasing use of biologic treatment in RA patients today.

However, RA registries themselves in our opinion are prone to selection bias by over-representing patients with a more severe disease course in the long-term follow-ups: RA patients with a milder disease course, as well as patients who were falsely diagnosed with RA, are more likely to return into the treatment of family practitioners and, thus, to be lost to follow up.

## 5. Conclusions

Despite available biologic treatment and achievement of satisfactory control of disease activity in the majority of patients in our study, RA remains a destructive disease with a high impact on functionality over the years. In order to further investigate the long-term outcomes of RA in relation to disease duration and specific treatment strategies, longitudinal studies are needed.

## Figures and Tables

**Table 1:** Patient characteristics.

Disease Duration (Years)	Number (n)	Age (Years, Mean)	Female * (%)	BMI * (kg/m^2^, Mean)	Pos. Fam. History * (%)	RF Pos. * (%)	ACPA Pos. * (%)	Rheumatoid Nodules * (%)	Extra-art. Manifest * (%)	MTX Pre-exposed * (%)	TNF Pre-exposed * (%)
all	7850	59.9	74.4	26.0	20.6	70.8	62.2	22.7	72.9	81.9	57.7
<5	2535	57.0	69.2	26.2	15.0	60.8	54.4	7.1	52.3	83.1	42.4
≥5–10	1990	58.8	74.2	26.4	21.5	69.6	63.9	15.6	61.8	84.1	61.8
≥10–15	1250	60.8	76.7	26.2	22.1	75.6	66.5	26.3	81.1	83.5	65.2
≥15–20	788	62.2	77.0	25.7	25.4	79.3	64.0	36.3	84.5	81.2	69.3
≥20–25	500	63.3	79.2	25.5	25.6	81.1	73.2	41.1	85.7	75.1	67.2
≥25–30	325	65.3	81.8	24.8	24.3	85.5	74.7	51.5	86.3	76.3	67.8
≥30–40	333	66.5	82.9	25.2	26.7	79.6	70.2	50.8	91.0	77.0	64.4
≥40	129	71.5	86.0	24.9	31.0	84.3	69.4	56.7	66.7	66.7	62.7

* analysed on patients with available data; ACPA: anti citrullinated peptide antibodies; RF: rheumatoid factor; MTX: methotrexate; TNF: tumour necrosis factor; BMI: body mass index; n: number; Pos: positive; Fam.: family.
